# The Role of Regular Eating and Self-Monitoring in the Treatment of Bulimia Nervosa: A Pilot Study of an Online Guided Self-Help CBT Program

**DOI:** 10.3390/bs7030039

**Published:** 2017-06-26

**Authors:** Sarah Barakat, Sarah Maguire, Lois Surgenor, Brooke Donnelly, Blagica Miceska, Kirsty Fromholtz, Janice Russell, Phillipa Hay, Stephen Touyz

**Affiliations:** 1 School of Psychology, University of Sydney, Sydney, NSW 2006, Australia; stephen.touyz@sydney.edu.au; 2 Centre for Eating and Dieting Disorders, Boden Institute, University of Sydney, Sydney, NSW 2006, Australia; servicedevelopmentofficer@gmail.com (S.M.); bm405@uowmail.edu.au (B.M.); kfromholtz@gmail.com (K.F.); 3 Department of Psychological Medicine, University of Otago at Christchurch, Christchurch 8140, New Zealand; lois.surgenor@otago.ac.nz; 4 Sydney Local Health District, Sydney, NSW 2006, Australia; brooke.donnelly@sswahs.nsw.gov.au; 5 School of Medicine, University of Sydney, Sydney, NSW 2006, Australia; janice.russell@sydney.edu.au; 6 Translational Health Research Institute, School of Medicine, Western Sydney University, Sydney, NSW 2751, Australia; p.hay@westernsydney.edu.au

**Keywords:** bulimia nervosa, online treatment, self-monitoring, regular eating, cognitive behavioural therapy, objective binge episodes, purging

## Abstract

*Background*: Despite cognitive behavioural therapy (CBT) being regarded as the first-line treatment option for bulimia nervosa (BN), barriers such as its time-consuming and expensive nature limit patient access. In order to broaden treatment availability and affordability, the efficacy and convenience of CBT could be improved through the use of online treatments and selective emphasis on its most ‘potent’ components of which behavioural techniques form the focus. *Method:* Twenty-six individuals with BN were enrolled in an online CBT-based self-help programme and 17 completed four weeks of regular eating and food-monitoring using the online Food Diary tool. Participants were contacted for a weekly check-in phone call and had their bulimic symptom severity assessed at five time points (baseline and weeks 1–4). *Results*: There was a significant decrease in the frequency of self-reported objective binge episodes, associated loss of control and objective binge days reported between pre- and post-treatment measures. Significant improvements were also observed in most subscales of the Eating Disorder Examination-Questionnaire. *Conclusion*: This study provides encouraging preliminary evidence of the potential of behavioural techniques of online CBT in the treatment of BN. Online therapy with this focus is potentially a viable and practical form of treatment delivery in this illness group. These preliminary findings support the need for larger studies using control groups.

## 1. Introduction

Therapist-led cognitive behavioural therapy (CBT) currently forms the most empirically validated treatment for bulimia nervosa (BN), and accordingly is widely recommended as the first line treatment for adults with BN [1,2]. However, the empirical support which this “gold-standard” treatment enjoys is challenged by the statistic that on average only 23.2% of eating disorder (ED) sufferers actually access treatment [3], with this figure substantially lower in some urban and rural regions with no specialist ED services [4]. Importantly, the quality of CBT delivered in the community is inconsistent. Tobin, Banker, Weisberg and Bowers [5] report that only 6% of clinicians adhere to an evidence-based manualised version of CBT for eating disorders (EDs), concluding that therapist ‘drift’ from CBT is the norm rather than the exception [6]. Moreover, CBT for BN is a specialist treatment, which for a routine delivery costs US$6000.00 per individual case [7], with the Australian healthcare system rebating less than half of the CBT sessions for a routine case treated by a psychologist [4]. 

Modification of CBT-BN structure and delivery is vital to ensure treatment is accessible [8]. Broadening the format of CBT delivery beyond the current therapist-led structure to an online, self-help program will offset the shortage of ED specialist clinicians. Additionally, overcoming the lengthy nature of CBT-BN can be achieved through investigation of the most powerful therapeutic components of CBT-BN, of which behavioural techniques are a promising candidate [9].

### 1.1. Treatment Delivery Formats to Increase Access

In order to address barriers to accessing treatment, a number of alternative treatment formats have evolved. These are briefly reviewed.

#### 1.1.1. Guided Self-Help

Various forms of guided self-help for BN have been available for several decades now, allowing a body of research to have evolved assessing its effectiveness. People who receive professional therapeutic support throughout their self-help treatment program display superior treatment outcomes to those independently engaging in self-help, producing equivalent outcomes to therapist led CBT for one-third of BN patients [10]. Accordingly, the addition of monthly thirty-minute guidance sessions for patients’ observing Fairburn’s *Overcoming Binge Eating* self-help manual [11] raised symptomatic improvement from 25% for those receiving no guidance, to 36% for telephone guidance and 50% for face-to-face guidance [12]. Supervision requirements for self-help treatment options are one-fifth of that required for a complete CBT course [13], advocating for a “stepped-care” approach which allows for the appropriate allocation of therapeutic skills according to one’s clinical severity [14,15]. 

#### 1.1.2. Online Self-Help

The successful application of CBT’s primary principles into recent technological advancements has been demonstrated across a range of formats including online programs [16,17,18,19,20,21], email correspondence [22,23], text messaging [24] and CD-ROM programs [25]. Pretorius et al. [19] recently evaluated a web-based CBT intervention consisting of eight 30–40 min interactive online sessions for a sample of 101 patients diagnosed with BN or eating disorder not otherwise specified (EDNOS) with bulimic features. In addition to weekly email support, completion of the eTherapy program resulted in a significant improvement in the frequency of objective binge episodes (OBEs), purge episodes and global Eating Disorder Examination (EDE) [26] maintained at the six-month follow up. Additional support for eTherapy has been provided by a study of 75 BN or EDNOS patients of whom 25.8% displayed abstinence from bulimic behaviours following engagement in an eight-session online CBT-based program [21]. These findings are comparable to the 20% and 30% post-treatment abstinence from bulimic behaviours observed following the use of manual-based CBT self-help books and face-to-face CBT treatment, respectively [10,14]. 

Self-monitoring via a technological device upholds numerous benefits to traditional pen and paper methods, including in-built reminders to prompt higher completion, more ecologically valid data, real-time monitoring and personalised, immediate feedback on one’s entries [27,28]. The recent popularity of a smartphone application for ED self-monitoring, known as ‘Recovery Record’, has seen 108,000 downloads across a two-year period, of which the majority of users logged their daily meals on the application for a thirty-day period [28]. However, Walsh and colleagues [27] found that while people with EDs viewed the idea of online monitoring favourably, they did not experience any benefit in practising the technique themselves. 

### 1.2. Behavioural Components of CBT

Despite the extensive number of clinical trials assessing CBT as an entire treatment package, little empirical evidence exists on the effectiveness of CBT’s individual therapeutic techniques [29]. Component analyses of CBT treatment for depression and anxiety demonstrate that therapy consisting purely of behavioural techniques produces equivalent outcomes to therapy involving both behavioural and cognitive techniques [30]. Moreover, in a comparative treatment trial for BN, CBT produced a greater shift in patients’ distorted attitudes regarding shape, weight and extreme dieting as compared to a simplified behavioural version of CBT, yet was equivalent in all other treatment outcomes [31]. Key behavioural components of CBT for BN are briefly reviewed below.

#### 1.2.1. Self-Monitoring 

Self-monitoring is one of the first behavioural tasks introduced in CBT whereby patients record a target behaviour and any associated antecedents as they occur within their natural context [32]. Self-monitoring allows the clinician and patient to jointly evaluate problematic behavioural patterns, work to reduce them and can spark reactive effects due to the inherently reinforcing or punishing nature of self-records [32]. 

In CBT-BN, daily self-monitoring is introduced in the first session and requires patients to record their food and drink intake, including binge or compensatory behaviours, time, meal type and associated feelings at each meal [33]. A number of experts credit self-monitoring as the most powerful therapeutic intervention in the treatment of BN [9,34]. In support of this, investigations of the temporal effectiveness of CBT reveal the largest reduction in key bulimic behaviours occurs within the first four to six weeks of treatment, while self-monitoring and regular eating are being introduced, and is the best predictor of short and long term treatment outcomes for BN patients [35,36,37,38,39]. 

Latner and Wilson [9] conducted one of the very few studies examining the effect of self-monitoring in BN treatment. Following six to seven days of food-monitoring, 30 individuals diagnosed with either BN or binge eating disorder (BED) reported a significant decrease in OBEs. It has also been found that in addition to a reduction in OBEs, self-monitoring produces a simultaneous increase in subjective binge episodes (SBEs). Such a response was termed ‘binge drift’, with Hildebrandt and Latner [34] claiming that although food monitoring allows for greater awareness of one’s eating behaviours, it may not target the destructive thoughts and negative affect, characteristic of the loss of control (LOC) experienced during binges. Other studies have examined the efficacy of self-monitoring alone in small or non-clinical samples, also suggesting its effectiveness [40,41]. 

#### 1.2.2. Regular Eating: The Three-Hour Rule

Paired with food monitoring is another behavioural activity known as the three-hour rule whereby patients are instructed to consume three planned meals and two or three planned snacks daily to ensure food deprivation does not exceed three to four hours [42,43]. Regular eating interrupts the heavy dietary restriction practiced by individuals with BN, reducing vulnerability to psychological and physical triggers for a binge [44]. 

Despite emphasis on regular eating as a fundamental component of CBT for BN [45], there has been limited empirical investigation into its effectiveness as a singular component. Shah, Passi, Bryson and Agras [46] reported the consumption of three meals and one snack daily resulted in the highest abstinence from binge eating and purging in a sample of 158 BN patients receiving either CBT or interpersonal psychotherapy (IPT). Additionally, high adherence to regular eating has also been shown to lower weekly binge frequency in a sample of 38 university students presenting with regular binge eating [44]. 

The current study endeavours to expand upon the preliminary research examining self-monitoring and regular eating to investigate their joint effectiveness in a modified CBT treatment program. In an attempt to enhance the efficacy of online CBT and consequently improve treatment outcomes for people with BN, the current study explores the therapeutic effectiveness of food monitoring and the “three-hour rule” via an online, low-intensity CBT program known as Binge Eating eTherapy (BEeT), recently developed by the Centre for Eating and Dieting Disorders (CEDD) at the University of Sydney. 

Firstly, it was hypothesised that following Stage 1 low-intensity CBT (online self-monitoring and “three-hour rule” training), participants will display a significant reduction in OBEs, LOC and compensatory behaviours. Secondly, in line with prior findings of ‘binge drift’, we hypothesise an increase in SBE pre-post treatment measures. 

BN possesses the highest psychiatric comorbidity of the ED’s [47], with up to 94.5% of sufferers meeting criteria for at least one DSM-IV disorder axis I diagnosis [48] and evidence suggests this comorbidity can be linked to more severe BN psychopathology [49,50] and symptom persistence [51]. For this reason, consideration of these factors was also considered pertinent in the current study. Specifically, we expected that a preliminary exploratory analysis of participants with comorbid mood disorders and/or severe bulimic behaviours in this cohort will display lower treatment compliance and poorer therapeutic outcomes.

## 2. Materials and Methods

### 2.1. Participants

Participants were recruited from the general Australian community using online advertisements on health websites, social media announcements, referrals from health professionals and paper advertisements placed on the grounds of the University of Sydney. The study was approved by the Sydney Local Health District Ethics Review Committee, Royal Prince Alfred Hospital Zone (Ethics Approval Number: X14-0302).

**Figure 1:**
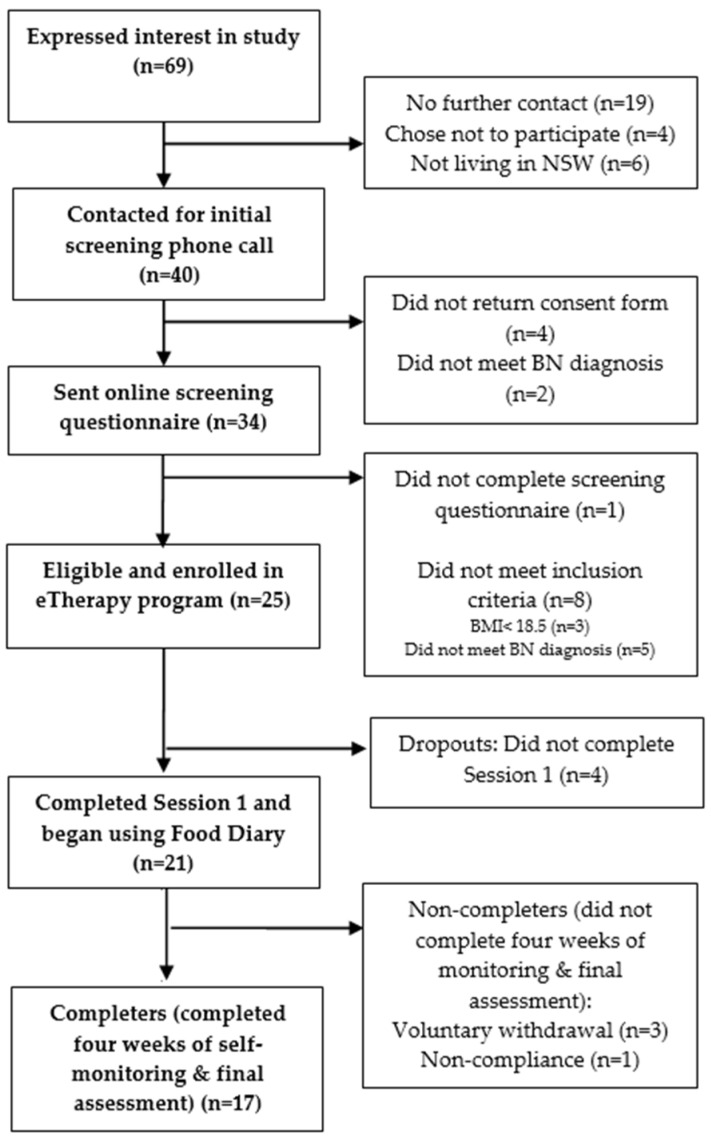
Flowchart of study participants.

Of the 69 individuals who expressed interest, 26 females entered the study (See Figure 1). Participants were eligible for this study if they were aged between 16 to 65 years of age and met a full DSM-5 [52] diagnostic criteria for BN (purging or non-purging type). Participants below 18 years of age were required to provide parental consent. As assessed by the Eating Disorder Examination Questionnaire (EDE-Q) [53], participants must have engaged in OBEs and inappropriate compensatory behaviours (inclusive of vomiting, excessive laxative or diuretic use, extreme exercise or severe dietary restriction) at least once per week in the preceding 28 days from when the questionnaire was taken. The presence of such behaviours across the preceding three months was confirmed by a senior clinical psychologist with experience in treating ED patients who discussed symptom presentation with the patient in a thorough phone interview. As part of the interview, participants’ self-evaluation due to shape and weight, as assessed on the EDE-Q, was also confirmed. 

Exclusion criteria included a body mass index (BMI) below 18.5 and current participation in a CBT psychological treatment specifically for their eating disorder. Individuals engaging in general psychological or pharmacological treatment were not excluded from the study, neither were participants with a comorbid psychiatric disorder. If a safety plan and regular monitoring of risk could not be established for participants with self-harm and suicide behaviours, they were excluded from the study. Monitoring of risk included follow-up phone calls if participants’ displayed thoughts of self-harm or suicide in their weekly questionnaires or Food Diary entries. In this phone call, the severity of these thoughts was clarified and regular contact of the participant with an informed health professional was confirmed. Previous or acute active suicidality or self-harm behaviours did not obviate inclusion. 

### 2.2. Materials

#### 2.2.1. Binge Eating eTherapy Program (BEeT)

The BEeT program consists of ten, one-hour interactive, multi-media sessions employing low-intensity CBT delivered by a pre-recorded therapist. Session 1 addresses the regular eating according to the “three-hour rule” and self-monitoring of eating using the online Food Diary. Access to the Food Diary tool was provided upon completion of Session 1 of the online program. Specifically, participants were educated on the impact of severe dietary restriction and how “delayed eating” is linked to urges to binge. Participants were encouraged to offset this by structuring their eating around three planned meals and two to three planned snacks daily.

The Food Diary tool is based upon the self-monitoring procedure for recording of eating behaviours as specified in most eating disorder therapies, including all existing CBT programs for BN [42,43,54,55]. Participants were to log a separate diary entry for each meal recording the following details: meal type, type and quantity of food and beverage consumed, the time at which intake occurred, whether the meal was considered to be a binge or over-eating, any urge to binge, type and quantity or length of any compensatory behaviours and associated events or feeling. On participants’ entry of their dinner record in the diary, end of day on-screen feedback is provided on the “three-hour rule” and on the presence of binges across that day. Importantly, the feedback encouraged participants to continue practising the behavioural activities. 

#### 2.2.2. SMS Reminders 

A daily SMS is sent to participants’ mobile phones at approximately 9 a.m. as a reminder to record their meals using the Food Diary tool throughout the day. An additional evening SMS is sent at approximately 6 p.m. to participants who had not completed a Food Diary entry for the preceding two days to prompt reengagement.

### 2.3. Psychometric Measures

All the psychometric measures listed below formed part of the pre- and post-treatment maxi eScreens. The mini eScreen weekly assessments consisted of a shortened version of the EDE-Q, the Kessler Psychological Distress Scale (K10) and the self-harm and suicidality risk assessment. All psychometric measures were self-report assessments administered online. There exists a high correspondence between the psychometric measures delivered using online and written formats [56].

#### 2.3.1. Eating Disorder Examination Questionnaire (EDE-Q)

The 16th edition of the EDE-Q [53] is a 30-item self-report version of the interview-delivered EDE [26]. The EDE-Q examines the behavioural features of one’s ED, including the frequency and days of OBEs and SBEs as well as the frequency of purging, laxative use and excessive exercise over the preceding 28 days. The EDE-Q also contains four subscales (Shape Concern, Weight Concern, Eating Restraint, and Eating Concern) assessing the cognitive and emotional aspects of the ED, which utilise a seven-point Likert scale ranging from scores of 0 to 6, with higher scores indicative of more severe symptomology. 

The EDE-Q was used to validate a diagnosis of BN and determine the severity of patients’ bulimic behaviours, with frequency of binging and compensatory behaviours constituting the primary outcome measures. The EDE-Q upholds good reliability (Cronbach’s α = 0.90) [57].

#### 2.3.2. Kessler Psychological Distress Scale (K10) 

The K10 [58] is a screening tool used to monitor psychological distress experienced by patients and comprises of ten questions, each consisting of five response options, which assess the degree of negative emotionality experienced across the past 28 days (pre- and post-treatment questionnaires) or 7 days (weekly questionnaires). The K10 has good reliability (Cronbach’s α = 0.93) [58].

#### 2.3.3. Eating Disorder Quality of Life Questionnaire (EDQOL)

The EDQOL [59] consists of 25-items examining the quality of life experienced by individuals suffering with an ED. Four subscales assess the impairment upon four primary domains including Psychological, Physical/Cognitive, Work/School and Financial domains. The EDQOL has good reliability (Cronbach’s α = 0.94) [59]. 

#### 2.3.4. Three-Factor Eating Questionnaire (TFEQ)

The TFEQ [60] consists of three subscales assessing dietary restraint, disinhibition or loss of control over eating and hunger perception. The self-report assessment consists of 36 items with a yes/no response option, 14 items using a 1–4 response scale and a single vertical rating item. 

#### 2.3.5. General Information and Demographics 

This series of questions concerns the general demographic information including age, gender, occupation, cultural background/ethnicity and setting of residence.

#### 2.3.6. General Mental Health

This series of questions gathers information regarding participants’ current primary and secondary mental health concerns, such as eating/weight issues, anxiety, stress, depression or substance/alcohol issues, whether or not these mental health concerns are being treated and by which mental health service, such as psychiatrist, psychologist, mental health nurse, social worker, counsellor, medical doctor or self-help book, and the type of treatment they are accessing, such as CBT, general counselling, hypnosis, antipsychotics or antidepressants.

#### 2.3.7. Self-Harm and Suicidality Risk Assessment

This assessment reviews the history of participants’ suicidal and self-harming thoughts and actions. It explores whether participants have had thoughts of suicide, attempted suicide or tried to injure or harm themselves in the previous 12 months or 28 days prior to completing the assessment. 

### 2.4. Procedure

**Figure 2:**
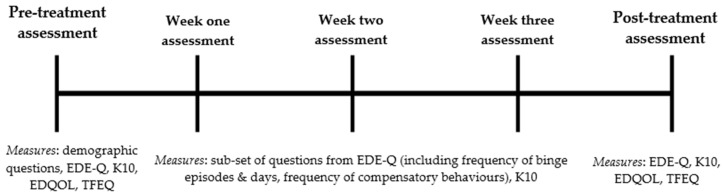
Schematic of the timing and sequence of assessments and included measures. Note: Week one, week two and week three assessments were identical. EDE-Q = Eating Disorder Examination-Questionnaire; K10 = Kessler Psychological Distress Scale; EDQOL = Eating Disorder Quality of Life Scale; TFEQ = Three Factor Eating Questionnaire.

A brief telephone assessment screened participants for suitability; those eligible completed written informed consent and were then administered a comprehensive online assessment. Participants were instructed to complete Session 1 of the program and upon completion to immediately begin recording subsequent meals and episodes of binging and/or compensation in the online Food Diary for the following 28 days (See Figure 2).

On the 7th, 14th and 21st day following their first Food Diary entry, participants completed a mini eScreen questionnaire and received a 15- to 20-min check-in phone call from a research assistant trained by the CI (clinical psychologist). Each phone call was guided by a standardised set of questions and feedback developed by a clinical psychologist (S.M.) with over 15 years of experience using CBT for BN. Obstacles hindering participants’ ability to adopt the Food Diary and three-hour rule were discussed, with a member of the treatment team presenting solutions in accordance with key CBT techniques of psychoeducation and positive reinforcement. Participants were sent an email the day prior to the scheduled phone call prompting completion of the mini eScreen questionnaire, followed by reminder text message on the day of the phone call. 

On the 28th day of using the Food Diary, participants completed the final comprehensive online assessment (maxi eScreen) and received a final check-in phone call. Participants then gained access to remaining components of the BEeT program to engage in a pure self-help format independent of the study protocol. 

### 2.5. Statistical Analysis

The data were cleaned and inspected for normality and baseline features of participants were examined. The statistical analysis was conducted in several stages, focusing on compliance then symptom outcome variance. First, attrition and compliance of participants with the treatment program was examined using the variables of mean number of days a Food Diary entry was made and the average number of daily Food Diary entries. 

Secondly, linear regression analysis was used to test the statistical significance of differences between the pre- and post-treatment dependent variables of OBE frequency, OBE days, SBE days, LOC frequency, purge frequency, laxative frequency, exercise frequency, EDE-Q subscale scores, global EDE-Q score, global EDQOL score and global TFEQ score. Comparisons between groups were based upon a two-tailed Bonferroni-corrected α of 0.0036 (0.05/14). Linear regression analyses and pairwise comparisons were used to assess changes in OBE frequency, OBE days, purge frequency, laxative frequency, SBE frequency, dietary restriction and the number of days of Food Diary entries between the week one, two and three measures. 

Finally, in order to determine if differences in participants’ clinical and demographic features at baseline were associated with their treatment compliance, bulimic symptom severity and bulimic symptom improvement, these three outcome variables were converted into binary variables. 

Treatment compliance variables (treatment completion, days of Food Diary entries, number of Food Diary entries) and treatment outcome variables (pre- to post-treatment change in binge frequency, purge frequency, laxative frequency, excessive exercise frequency, dietary restraint score) were converted into three binary distributions with the reference point (or cut-off) specified at 50%, 75% and 90% of the original variable value. Three distributions were created as a precautionary measure in the absence of any prior convention from previous BN treatment studies regarding the conversion of continuous dependent variables into binary variables. 

A series of paired t-tests were used to examine differences between the binary variable levels in terms of the continuous variables of age and K10 score. Similarly, a Fisher’s exact test examined differences between binary variable levels in terms of the categorical predictor variables of secondary mental health concerns, active/past suicidality and active/past self-harm. Analyses were conducted using the SPSS for Mac OS X version 22.0.

## 3. Results

### 3.1. Participant Characteristics 

The mean age of the 25 female participants enrolled in the eTherapy program was 30.24 years (range 16–47 years, *SD *= 9.37) and the mean BMI was 25.1 (range 18.8–50.7, *SD *= 6.88). Only one participant was below 18 years of age. The sample included participants with normal weight BN and overweight BN. All participants met the DSM-5 criteria for BN at baseline [52]. Five participants (20.0%) satisfied the DSM-5 criteria for BN, non-purging type [52], the remainder met the purging type category. A high proportion of participants reported comorbid anxiety (32.0%) or depression (40.0%) as a secondary mental health concern. No participants were excluded on the basis of their self-harm or suicidality assessment. Nineteen participants (76.0%) reported engaging in another form of treatment at baseline. The most commonly reported treating professional was a psychologist or psychiatrist (56.0%), followed by GP (24.0%) and dietitian (12.0%). The sociodemographic variables of participants as reported at baseline are displayed in Table 1. 

### 3.2. Attrition and Compliance

Of the 25 participants enrolled in the online program, four participants (16.0%) did not complete Session 1 and therefore did not access the Food Diary tool (dropouts). Four participants (16.0%) failed to complete the four weeks of monitoring and the final assessment (non-completers) and 17 participants (68.0%) engaged in the four-weeks of treatment and completed the final assessment (completers). The non-completers engaged in the Food Diary tool for an average of 18.8 days (*SD *= 2.06), that is approximately 2.5 weeks, prior to disengaging from the treatment program. 

On average, the 17 completers logged at least one entry in the online Food Diary for 87.1% of the possible 28 days (range: 16–28 days, *M *= 24.4, *SD *= 4.76). The most common number of daily Food Diary entries made by all participants was five per day. There was a significant difference in the mean number of days of self-monitoring (indexed by at least one Food Diary entry) between the four weeks of treatment, Wald χ^2^ (3, *N* = 17) = 15.03, *p *= 0.002. Specifically, there was a significant decrease in the mean number of days monitored between week one (*M *= 6.7, *SD *= 0.86) and week two (*M *= 6.1, *SD *= 1.52, *p *= 0.042); week three (*M *= 6.06, *SD *= 1.89) and week four (*M *= 5.47, *SD *= 2.04, *p *= 0.05) and week one and week four (*p *= 0.002). Generally, the mean number of days monitored decreased each week, although at the end of treatment (week four) the average was still approximately 5.5 days of the 7. Participants’ (*n* = 17) regularly monitored their food intake (indexed by five or more Food Diary entries) for an average of 21.2% of the possible 28 days (range: 0–28 days, *M *= 5.9 days, *SD *= 8.00). 

Due to scheduling constraints, 13.2% of weekly phone calls were not completed at the exact seven-day mark. On average, these phone calls occurred 2.0 days (range: 1–8 days, *SD *= 2.35) later than the scheduled date and five phone calls did not take place. Moreover, 26.4% of the weekly questionnaires were not completed on time at the 7-day mark and were delayed by an average of 2.83 days (range: 1–11 days, *SD *= 2.81). 

### 3.3. Treatment Outcomes 

Table 2 provides the means and standard deviations for all dependent variables at each assessment period, as well as regression analysis results. Participants (*n* = 17) displayed a significant pre-post treatment decrease in the mean frequency of objective binge episodes, Wald χ^2^ (1, *N* = 17) = 21.62, *p *< 0.00, mean number of days participants’ experienced OBEs, Wald χ^2^ (1, *N* = 17) = 12.99, *p *< 0.001 and frequency of perceived loss of control experienced during OBEs, Wald χ^2^ (1, *N* = 17) = 12.47, *p *< 0.001. Pairwise comparisons revealed a significant increase in the mean number of objective binge days experienced in week one (*M *= 2.7, *SD *= 2.03) to week two (*M *= 3.5, *SD *= 2.07), *p *= 0.006. 

There was no significant change in the number of days participants experienced subjective binge episodes between pre- and post-treatment measures, Wald χ^2^ (1, *N* = 17) = 0.704, *p *= 0.401.

Using the Bonferroni correction, the decrease in the pre-post treatment frequency of purging episodes was no longer significant, Wald χ^2^ (1, *N* = 13) = 4.30, *p *= 0.038. No significant differences were found in other compensatory behaviours of laxative use and excessive exercise.

There was a significant decrease from baseline to post-treatment in the mean global score on the EDE-Q, Wald χ^2^ (1, *N* = 17) = 12.86, *p *< 0.001, as well as the mean scores on three EDE-Q subscales including dietary restraint, Wald χ^2^ (1, *N* = 17) = 10.75, *p *= 0.001, eating concern, Wald χ^2^ (1, *N* = 17) = 10.18, *p *= 0.001 and shape concern, Wald χ^2^ (1, *N* = 17) = 9.92, *p *= 0.002. 

The results of the Wilcoxon signed rank test (non-parametric sensitivity analysis) supported the respective statistical significance and non-significance of the preceding regression analyses examining the difference in bulimic symptomology between pre- and post-treatment measures. 

### 3.4. Predictors of Outcome and Dropout 

The Fisher’s Exact Test revealed a significant association between the participants’ level of bulimic severity and their degree of improvement in binge frequency, *p *= 0.002. Participants who displayed a 50% or greater reduction in binge frequency all were classed as less severe in bulimic presentation (defined by an average of 1–7 compensatory behaviours per week). In contrast, the majority (77.8%) of participants who displayed less than 50% reduction in binge frequency were classified as more severe in bulimic presentation. 

Bulimic symptom severity was also associated with dietary restraint, *p *= 0.050. A statistical trend emerged whereby a larger number of participants who displayed a 50% or greater reduction in dietary restraint scores, had low bulimic severity (87.5%) as compared to high bulimic severity (12.5%). Conversely, more participants with high bulimic severity (66.7%) displayed less than 50% reduction in restraint scores as compared to those with low bulimic severity (33.3%).

Overall, treatment compliance and degree of improvement in compensatory behaviours were not significantly associated with participants’ age, secondary mental health concern, K10 score, active/past suicidality, active/past self-harm and bulimic symptom severity. 

## 4. Discussion

The findings of this study provide preliminary support for the food monitoring and regular eating techniques of CBT delivered online. Four weeks of guided, online stage 1 CBT resulted in a significant decrease in the frequency of OBEs, number of OBE days and frequency of associated LOC from baseline to post-treatment measures. There was also a significant decrease in both global EDE-Q scores and the EDE-Q subscales of dietary restraint, eating concern and shape concern. No change was observed in frequency of SBEs and compensatory behaviours including purging, laxative use and excessive exercise. 

### 4.1. Attrition and Compliance

The current study has a considerably lower dropout rate (16%) than other online CBT studies (35% to 82%) [20]. Treatment compliance remained high with the Food Diary being entered at least once for an average of 87.1% of the possible 28 days. The food-monitoring compliance reported by previous studies is varied, ranging from 100% compliance for six to seven days of self-monitoring [9,34] to 46% compliance for 56 days of self-monitoring [41] The compliance achieved by the present study fits within the range reported by others and suggests that 28 days of food-monitoring may be an appropriate compromise between harnessing the technique’s therapeutic potential and maintaining motivation to engage. Furthermore, many participants reported that the repetitive and tiresome nature of food monitoring was largely offset by the motivating nature of the weekly phone calls, with such anecdotal feedback suggesting an important role for therapeutic contact treatment compliance. However, due to the absence of appropriate control groups, the role of regular therapeutic contact and digital access to the Food Diary in producing heightened treatment compliance is yet to be elucidated. 

Inaccuracies in the logging of meals in the Food Diary prevented accurate evaluation of participants’ compliance with the “three-hour rule”. The absence of such valuable information is due to the inaccurate recording of meal time or meal type, with most participants not adjusting the default settings of 7:00 a.m. and Breakfast when entering in the Food Diary. Consequently, an analysis of this data was not conducted. We recommend future studies adopt more stringently formatted systems to ensure accurate timing of meals is recorded [44]. 

### 4.2. Treatment Outcomes

The brief four week intervention achieved key symptom improvement comparable to the treatment outcomes of considerably longer online CBT self-help programs lasting for two to four months on average [16,17,18,19,21,61]. The preliminary results here contradict a recent review reporting that people with EDs do not find self-monitoring beneficial in practice [27] and rather supports the potential for concise, online self-monitoring programs. It is possible, however, that the observed improvements may possess short-term therapeutic effect, necessitating self-monitoring be examined over an extended time period which may cause the task to become burdensome. 

The absence of a significant reduction across compensatory behaviours, despite an observed improvement in binging measures, may be due to several features of the current study. These include the short intervention period of four weeks and the small subset of participants having reported engaging in purging (*n* = 13), laxative use (*n* = 7) and excessive exercise (*n* = 11) as compensatory behaviours. Alternatively, given that the primary objective of food monitoring and the “three-hour rule” is to establish a regular eating pattern [42], it would appear logical for earlier change to be observed in binge frequency prior to compensatory behaviours. The paucity of evidence regarding the therapeutic effect of regular eating upon compensatory behaviours [46] necessitates future replication using larger sample sizes to confirm such suggestions. Additionally, considering early treatment response is known to predict short and long term BN treatment outcomes [38,39], the significant improvement observed in OBEs, LOC and dietary restraint following the use of only two behavioural techniques from Fairburn’s 20-week program [42] may be indicative of future improvement. 

Although participants’ displayed an overall reduction in the number of OBE days from pre- to post-treatment measures, there was an unexpected increase in OBE days from week one (2.65 days) to week two (3.53 days). Given that the week two questionnaire was completed at the mid-point of treatment, perhaps the observed trend represents participants’ initial motivation for recovery becoming later overpowered by the strength and habitual nature of their disordered eating behaviours. Alternatively, having people with BN closely review their egodystonic symptoms in the form of a Food Diary may prompt a decrease in mood and increase the likelihood of binges to regulate the distress and shame surrounding their eating behaviours [62]. More simply, participants may have developed heightened awareness regarding their behaviours in the second week of monitoring or perhaps documented their binges more truthfully once they became more comfortable with the treatment process. Further research is required to clarify whether an initial rise in OBE days is a typical BN treatment response or an anomaly of the current study.

Contrary to Hypothesis 2, no significant change was found in the number of days participants’ recorded experiencing SBEs, and this challenges Hildebrandt and Latner’s [34] ‘binge drift’ observation. The theoretical justification for ‘binge drift’ as the inability of self-monitoring to adjust dysfunctional cognitions [34] is also challenged by the observed decrease in the EDE-Q measures of attitudinal eating disorder psychopathology. Self-help CBT programs consisting of a longer two month treatment program have been unable to achieve such attitudinal change, similarly claiming that additional treatment time and greater focus upon cognitive elements are required [63,64]. Thus, the attitudinal improvement brought about by a considerably shorter behaviourally-based program alludes to suggestions that cognitive adjustments can be prompted by engaging in behavioural activities [30]. For example, persistence in implementing the “three-hour rule” allows for first-hand experience of the benefits of regular eating and provokes correction of certain fears or maladaptive cognitions regarding their shape. In a similar manner to the component analyses of CBT for depression [65], the ability of behavioural techniques to harness improvement in cognitive measures must be validated through direct comparison of the cognitive and behavioural components of CBT-BN. 

### 4.3. Predictors of Treatment Compliance and Outcome

Participants with a more severe clinical presentation of BN displayed a smaller improvement in both OBE frequency and dietary restraint as compared to participants with less severe BN symptomology. Differential treatment responses as a function of one’s clinical severity is mirrored in similar studies of guided self-help treatment [13,15] and fits within the “stepped-care” structure of treatment allocation [14]. Online stage 1 CBT appears to approach the therapeutic effectiveness of face-to-face treatment for a sub-set of less severe BN patients, whilst overcoming the barriers of cost, accessibility and duration of a treatment format, which may be unnecessarily excessive for their condition [8]. However, given the small sample size, it is possible that the statistical trend observed between dietary restraint and bulimic severity may represent a Type 2 error and requires replication in larger samples.

In contrast with existing literature on CBT treatment for BN [49,66,67], in this sample treatment compliance and bulimic symptom improvement was not significantly associated with baseline measures of depression or the presence of comorbid mood or anxiety disorders. The absence of such association may be due to the heavy concentration of behavioural techniques within the current study. Behavioural activities provide a practical and “hands-on” approach to treatment that is likely to enhance self-efficacy and offset the hopelessness brought upon by depressive traits and the bulimic symptoms [65,68,69]. Specifically, self-monitoring allows for direct observation of one’s efforts to adhere to the “three-hour rule” and provides objective evidence of their attempts. Taken together with the encouragement provided by a therapeutic assistant, behavioural activities provide direct, observable change in depressed participants which may shift their external locus of control [66] and offset poor treatment compliance. Such justification is supported by recent suggestions regarding the importance of motivation as a predictor of BN treatment success [70] and should be included as a baseline measure in future studies. Additionally, the equivalent treatment compliance and therapeutic effectiveness across participants aged between 16 to 47 years, once more broadens the scope of candidates suitable for this treatment. 

### 4.4. Strengths, Limitations and Future Research

The current study provides a novel contribution to the literature on BN treatment by offering preliminary pilot study evidence for the clinical effectiveness of an online, stage 1 CBT program. The BEeT program employed in the study is a highly structured and sophisticated program, professionally developed by team of ED specialists and constructed based upon relevant empirical research on CBT-BN. Additionally, therapeutic guidance was provided by a non-expert trained in how to deliver the behavioural components of CBT treatment, offering promise as a method that could be used broadly by non-experts. The present study improves upon methodological limitations in the existing literature [71] having recruited a pure clinical sample of BN patients. Additionally, all communication took place via email and the telephone, maintaining the largely anonymous nature of online treatment and increasing the accessibility for patients who are busy with work, study or carer commitments as face-to-face interviews were not required. 

Due to the relatively small sample size, these findings must be considered tentative until an adequately powered study is conducted. The absence of a waitlist control condition also limits the conclusions regarding the causal role of the treatment program and necessitates other robust research designs be conducted. A randomised controlled trial will also allow for comparison of online, stage 1 CBT against conventional CBT programs, with the presence of control arms providing further clarity regarding the role of therapeutic guidance techniques, such as reminder text messages and weekly phone calls, in provoking the greatest change in one’s behaviours. 

Given that 56% of participants reported receiving additional treatment, other than CBT, for their ED, it is possible that improvement observed may be partly attributed to their concurrent treatment program. However, seeing as all participants were engaged in their additional treatment prior to entering the current study, it appears that the self-monitoring and regular eating techniques enforced may have been instrumental in bringing about change.

Future research should control for concurrent treatment to isolate the effects of behavioural techniques and strengthen claims regarding its therapeutic effectiveness. Furthermore, considering previous research [72] suggests that the reactive effects of self-monitoring are limited to the time period for which the individual actively engages in self-monitoring, future studies should include a follow-up.

## 5. Conclusions

The present study provides preliminary evidence for the clinical effectiveness of brief behavioural self-monitoring and regular eating training (BEeT) delivered online which, if replicated in a larger sample, may result in the development of shorter, yet equally effective, forms of treatment. Concise programs would make treatment accessible for a much broader range of people with BN for whom their work or study commitments and financial constraints make it extremely difficult for them to engage in three months of one-hour weekly therapy sessions [14]. Additionally, the digital format of treatment delivery overcomes the barriers of accessibility and cost inherent in the treatment of EDs and which are of particular concern for rural and regional residents. The current findings provide the justification for a randomised controlled trial to elucidate the clinical benefits of emphasising behavioural techniques which form part of typical CBT-BN delivery by online modalities.

## Figures and Tables

**Table 1:** Baseline characteristics of participants (*n* = 25).

Participant Feature	Frequency (%)
Employed	60
Student	24
White/Caucasian	84
Asian	12
Metropolitan residence	64
Regional residence *	24
Rural residence	12
Comorbid anxiety	32
Comorbid depression	40
Comorbid substance/alcohol issues	16
Receiving concurrent treatment	76
Frequent alcohol consumption (4 or more times per week)	36
Past suicidality	60
Active suicidality (within previous 28 days)	20
Past self-harm	48
Active self-harm (within previous 28 days)	12

* NSW Health categorises regional residence as outside a major metropolitan city (e.g., Sydney) but not a rural location. Examples of regional locations include Wyong and Wagga Wagga.

**Table 2:** Treatment outcomes for Eating Disorder Examination-Questionnaire (EDE-Q), Eating Disorder Quality of Life Scale (EDQOL) and Three Factor Eating Questionnaire (TFEQ) Scores (*n* = 17).

Outcome	Pre-Treatment Means (*SD*)	Post-Treatment Means (*SD*)	*p* Value	Test Statistic Wald χ^2^ (df = 1)
**EDE-Q**				
Objective binge frequency	23.7 (16.63)	14.9 (12.55)	*p <* 0.001 *	21.62
Objective binge days	17.9 (6.17)	10.8 (9.31)	*p* < 0.001 *	12.99
Loss of control frequency	21.0 (12.01)	14.1 (13.87)	*p* < 0.001 *	12.47
Subjective binge days (*n* = 16)	9.7 (9.60)	7.4 (6.26)	*p* = 0.401	0.70
Purge frequency (*n* = 13)	20.4 (20.45)	14.4 (18.85)	*p* = 0.038	4.30
Laxative use frequency (*n* = 7)	11.9 (11.45)	9.3 (10.61)	*p* = 0.071	3.25
Excessive exercise frequency (*n* = 11)	9.5 (10.58)	6.5 (8.42)	*p* = 0.123	2.38
Dietary restraint	4.1 (1.14)	2.9 (1.84)	*p* = 0.001 *	10.75
Eating concern	4.1 (1.11)	3.0 (1.41)	*p* = 0.001 *	10.18
Shape concern	5.3 (0.72)	4.5 (1.25)	*p* = 0.002 *	9.92
Weight concern	5.0 (0.60)	4.4 (1.48)	*p* = 0.076	3.14
Global score	4.6 (0.55)	3.7 (1.31)	*p* < 0.001 *	12.86
**Other Outcomes**				
EDQOL global score	1.9 (0.71)	1.8 (0.865)	*p* = 0.564	0.33
TFEQ global score	34.5 (4.91)	34.7 (4.61)	*p* = 0.888	0.02

* Statistically significant (*p* < 0.0036). Comparisons based upon Bonferroni-corrected α of 0.0036 (0.05/14). Note: EDE-Q = Eating Disorder Examination-Questionnaire; EDQOL = Eating Disorder Quality of Life Scale; TFEQ = Three Factor Eating Questionnaire.
